# Neural Plasticity in Human Brain Connectivity: The Effects of Long Term Deep Brain Stimulation of the Subthalamic Nucleus in Parkinson’s Disease

**DOI:** 10.1371/journal.pone.0086496

**Published:** 2014-01-22

**Authors:** Tim J. van Hartevelt, Joana Cabral, Gustavo Deco, Arne Møller, Alexander L. Green, Tipu Z. Aziz, Morten L. Kringelbach

**Affiliations:** 1 Center of Functionally Integrative Neuroscience (CFIN), Aarhus University, Aarhus, Denmark; 2 Department of Psychiatry, University of Oxford, Oxford, United Kingdom; 3 Center of Brain and Cognition, Theoretical and Computational Neuroscience Group, Universitat Pompeu Fabra, Barcelona, Spain; 4 Institució Catalana de la Recerca i Estudis Avançats (ICREA), Universitat Pompeu Fabra, Barcelona, Spain; 5 Nuffield Department of Surgical Sciences, John Radcliffe Hospital, Oxford, United Kingdom; Florey Institute of Neuroscience and Mental Health, The University of Melbourne, Australia

## Abstract

**Background:**

Positive clinical outcomes are now well established for deep brain stimulation, but little is known about the effects of long-term deep brain stimulation on brain structural and functional connectivity. Here, we used the rare opportunity to acquire pre- and postoperative diffusion tensor imaging in a patient undergoing deep brain stimulation in bilateral subthalamic nuclei for Parkinson’s Disease. This allowed us to analyse the differences in structural connectivity before and after deep brain stimulation. Further, a computational model of spontaneous brain activity was used to estimate the changes in functional connectivity arising from the specific changes in structural connectivity.

**Results:**

We found significant localised structural changes as a result of long-term deep brain stimulation. These changes were found in sensory-motor, prefrontal/limbic, and olfactory brain regions which are known to be affected in Parkinson’s Disease. The nature of these changes was an increase of nodal efficiency in most areas and a decrease of nodal efficiency in the precentral sensory-motor area. Importantly, the computational model clearly shows the impact of deep brain stimulation-induced structural alterations on functional brain changes, which is to shift the neural dynamics back towards a healthy regime. The results demonstrate that deep brain stimulation in Parkinson’s Disease leads to a topological reorganisation towards healthy bifurcation of the functional networks measured in controls, which suggests a potential neural mechanism for the alleviation of symptoms.

**Conclusions:**

The findings suggest that long-term deep brain stimulation has not only restorative effects on the structural connectivity, but also affects the functional connectivity at a global level. Overall, our results support causal changes in human neural plasticity after long-term deep brain stimulation and may help to identify the underlying mechanisms of deep brain stimulation.

## Introduction

Deep brain stimulation (DBS) is a neurosurgical procedure that is increasingly used to alleviate the symptoms of a number of otherwise intractable disorders including Parkinson's Disease (PD), essential tremor, dystonia and chronic pain [1], [2]. DBS for PD has become well established since the 1990s with two main surgical targets, namely the subthalamic nucleus (STN) and the globus pallidus internal (GPi) [3], [4], [5], [6]. Recently, another target in the pedunculopontine nucleus (PPN) has also shown promise [7]. The PPN is a relatively new target in treating primarily gait and posture symptoms in PD. Recent studies and reviews show positive results [8], [9]. Some studies however report much less positive results [10], [11].

Although positive clinical outcomes have now been well established, little is known about the effects of long-term stimulation on brain structure in terms of grey and white matter connectivity and the underlying neural mechanisms. Some insight has come from a rat study using the 6-OHDA model of Parkinson’s disease showing that prolonged high frequency stimulation of the STN has a neurorestorative action [12]. This finding suggests that DBS can change brain connectivity, corroborating previous findings from a human case study which, using diffusion tensor imaging (DTI) in PD, has demonstrated that DBS in the pedunculopontine nucleus (PPN) has a restorative action and increases connectivity of the PPN with the cerebellum [13].

What is not yet clear is how DBS affects whole-brain connectivity. The current study describes the case of a patient with PD undergoing bilateral STN DBS surgery. In this rare case we were able to acquire both preoperative and five-month postoperative DTI from this patient, which allowed us to investigate the long-term effects of DBS for PD using brain connectivity measures and computational modelling. This study involves a rare case due to the safety concerns and major artefacts related to postoperative MRI and DTI data acquisition. This study aims to elucidate long-term effects of DBS on the topological properties of structural brain networks, investigate the restorative function of DBS and predict the dynamical impact of such structural alterations on resting-state functional networks.

To begin with the structural networks, using advanced graph theoretical measures we concentrate on measures of *nodal efficiency*. Nodal efficiency is related to shortest path length and is believed to characterize the ability of parallel information transfer and large-scale functional integration in brain networks [14]. Alterations in the nodal efficiency of structural brain networks in PD may be linked to the cognitive and behavioural problems occurring in the advanced stages of the disease. Therefore, the recovery of healthy nodal efficiency values after DBS could be indicative of an effective restorative process in PD mediated by DBS.

Local alterations in white-matter structural connectivity, as observed here in PD before and after DBS, can have significant impacts on the large-scale dynamics of the brain. As a matter of fact, several works have shown that the structural connectivity, revealed by DTI, strongly shapes the functional connectivity (FC) between brain areas during rest (measured as the temporal correlations of the blood-oxygen-level-dependent (BOLD) signal recorded with functional MRI (fMRI)) [15]. However, the relationship between anatomical and functional brain connectivity is not trivial, and computational models of large-scale neural dynamics are unique tools to explore this relationship [16], [17]. Importantly, models can be used to predict the effects of structural alterations on brain dynamics [18], [19], which is beyond reach on the experimental side, making models a unique tool for the comprehension of brain diseases.

To investigate the dynamical impact of the structural changes occurring in PD before and after DBS, we used a dynamic mean field model of spontaneous activity [16], [20]. The spontaneous dynamics obtained with the different structural connectomes, i.e. pre-DBS, post-DBS and healthy controls, was analysed in terms of stability and BOLD functional connectivity. In a previous work [20], it was found that the optimal fit of the model with healthy resting-state functional connectivity was obtained just before the bifurcation point, i.e. the point above which neural activity becomes unstable or chaotic, suggesting that the brain at rest operates at the edge of instability. In the current work, we observed that this bifurcation point was shifted in PD. Notably, it was found that DBS induced the recovery of the structural connectivity, so that the bifurcation point was shifted back towards healthy values. In addition, we compared the simulated BOLD functional connectivity obtained with the different connectomes with a typical FC from healthy controls [21], We found that, despite the shift of the bifurcation occurring in PD, the dynamics exhibits homeostasis, i.e. the optimal fit with the empirical data was always found just below the bifurcation threshold, independently of the structural connectome considered. Finally, the topological properties of simulated functional networks were analysed using measures from graph theory. Results indicate that DBS induces a topological reorganisation of functional connectivity, recovering its properties towards healthy values.

We predicted that long-term DBS concomitant with alleviation of some clinical symptoms would affect regions which are known to be connected with the STN and which change in PD. As such, we predicted changes in the frontal cortices, which have been shown to have high coherence with the STN in PD [5]. We also predicted changes in the olfactory system given that olfactory dysfunction is a common symptom in PD [22]. Prevalence estimates of olfactory dysfunction in PD vary between 70–90% of patients [23]. Yet, the importance of the olfactory system in PD lies not only in its high prevalence. Olfactory dysfunction could be the first sign of PD and appears approximately five years prior to the onset of any motor symptoms [24]. Hummel and colleagues [25] have found that DBS of the STN in patients with PD significantly improves odour discrimination when DBS is turned on. No effect however was found for odour detection threshold, indicating changes in higher order olfactory areas. Given these previous behavioural results investigating the effects of STN DBS on olfactory functioning in PD, we predicted a change in nodal efficiency in olfactory regions including the primary olfactory cortex as well as in the secondary olfactory cortices in the orbitofrontal cortex [26]. Finally, we predicted that the functional connectivity resulting from the computational modelling of the brain networks post-DBS would show more similar results to that of a healthy functional connectivity compared to the strongly PD networks in pre-DBS state.

## Methods

### Patient, Healthy Participants and Surgical Procedure

Diffusion tensor imaging (DTI) data were acquired preoperatively and five months postoperatively in a 45-year-old female patient who underwent DBS surgery for PD. The main troublesome symptoms were on/off fluctuations and troublesome dyskinesias. The patient received continuous DBS stimulation *on* and *off* over 5 months during which the stimulation parameters were optimised. The patient received a post-DBS DTI scan to plan a lead revision warranted by adverse side effects (emotional lability and tearfulness) from the STN stimulation. After DBS implantation the medication was initially continued with Pramipexole 0.7 one and a half tablets three times a day, Stalevo was reduced to 50 mg three times a day (from 150 mg in the morning, 50 mg twice a day and 100 mg in the evening) and Amantadine was stopped completely (from 100 mg twice a day). The patient was advised to return to her preoperative medication regime during the periods when the DBS was turned off. Right DBS lead titration resulted in improvement in rigidity. Left DBS lead titration was more problematic (possibly due to the suboptimal positioning of the electrode) resulting in side effects. Stimulation parameters changed during these 5 months due to fine tuning and titration of the DBS electrodes. The postoperative DTI scan was acquired for a DBS lead revision to the GPi.

Additional DTI data were acquired for 9 healthy participants (3 females, age range 22–40 years) on the same scanner as the PD patient. This study was approved by the National Research Ethics Service (NRES) committee South Central – Berkshire in Bristol. All healthy participants gave written informed consent. The individual in this manuscript has given written informed consent (as outlined in PLOS consent form) to publish these case details.

#### Surgical procedure

Electrodes for DBS were implanted in the subthalamic nucleus (STN) bilaterally. Before surgery, anatomical high-resolution T1 and T2 MRI scans with 1×1×1 mm voxel size were acquired to plan the electrode implant protocol. The Cosman–Roberts–Wells stereotactic frame was applied to the patient’s skull under local anaesthetic. For a detailed description of the surgical procedure, see Kringelbach and colleagues [27].

### Image Acquisition

#### Patient

All DTI data for the patient were acquired on a Philips Achieva 1.5 Tesla Magnet in Oxford. Diffusion weighted imaging was performed using a single-shot echo planar sequence with coverage of the whole brain. The scanning parameters were echo time (TE) = 65 ms, repetition time (TR) = 9390 ms, reconstructed matrix 176×176 and reconstructed voxel size of 1.8×1.8 mm and slice thickness of 2 mm. Furthermore, DTI data were acquired with 33 optimal nonlinear diffusion gradient directions (*b* = 1200 s/mm^2^) and 1 non-diffusion weighted volume (*b* = 0). The post-DBS DTI data was acquired with DBS turned off.

#### Healthy participants

Nine healthy participants were scanned with the exact same parameters on the same clinical scanner.

### Structural Brain Networks

**Figure 1:**
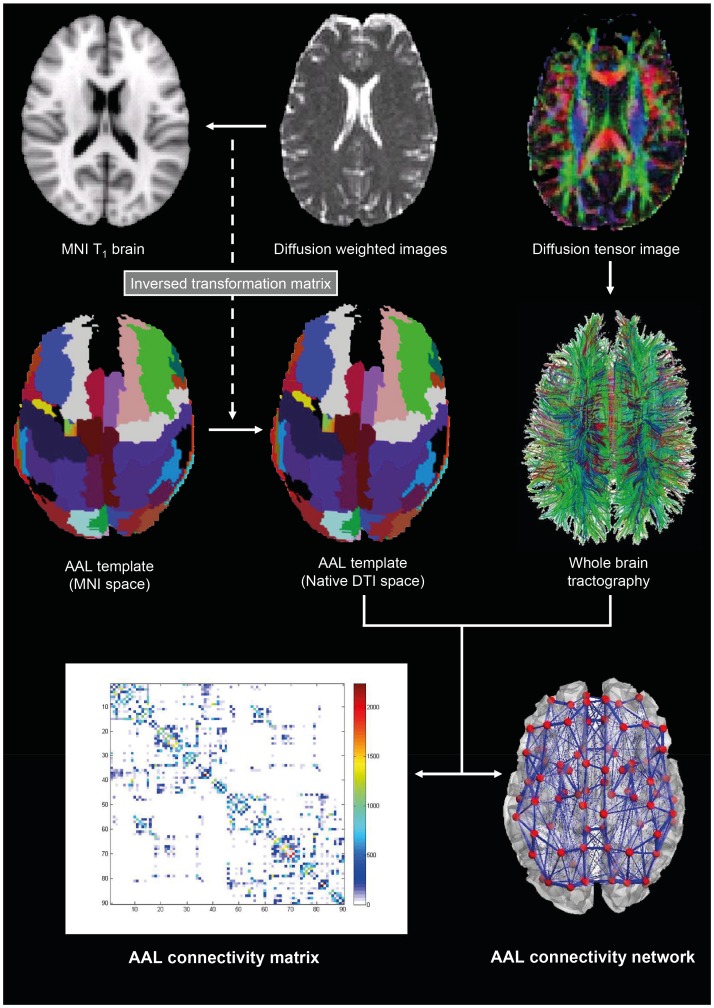
DTI and network construction based on the Automated Anatomical Labelling (AAL) parcellation [28].

The construction of structural brain networks consisted of a two-step process. First, the nodes of the network were defined using brain parcellation techniques. Secondly, the connections between nodes (i.e. edges) were estimated using probabilistic tractography (**Figure 1**). In the following we outline the details involved in each step.

#### 1) Brain parcellation

We used the Automated Anatomical Labeling (AAL) template to parcellate the entire brain into 90 cortical and subcortical regions (45 for each hemisphere), where each region represents a node of the brain network [28]. Additionally we incorporated the probabilistic mask of the STN from Forstmann and colleagues [29] resulting in a total of 92 cortical and subcortical regions (46 per hemisphere). The parcellation was conducted in the diffusion MRI native space.

We used the Flirt tool (FMRIB, Oxford) [30] to linearly coregister the b0 image in diffusion MRI space to the T1-weighted structural image. The transformed T1-weighted image was then mapped to the T1 template of ICBM152 in MNI space [31]. The resulting transformation was inversed and further applied to warp the Automated Anatomical Labeling (AAL) template [28] and the STN masks [29] from MNI space to the diffusion MRI native space, where interpolation using nearest-neighbour method ensured that the discrete labeling values were preserved.

Finally, a binary mask of the electrode lead in the post-DBS DTI data has been created and has subsequently been subtracted from the brain masks and data of the pre-DBS DTI data as well as from the DTI data of the healthy controls. Using this method we can minimise the effects of the electrode lead within the right hemisphere.

#### 2) Analysis of interregional connectivity

We used the FDT toolbox in FSL (version 5.0, http://www.fmrib.ox.ac.uk/fsl/, FMRIB, Oxford) to carry out the various processing stages of the diffusion MRI data. The initial preprocessing involved coregistering the diffusion-weighted images to a reference volume using an affine transformation for the correction of head motion as well as eddy current induced image distortion. Following this preprocessing, we estimated the local probability distribution of fibre direction at each voxel using the default bedpostx parameters of FSL v5.0 [32]. We then used the probtrackx algorithm allowing for automatic estimation of two fibre directions within each voxel, as specified in the previous step using parameter estimation from bedpostx, which can significantly improve the tracking sensitivity of non-dominant fibre populations in the human brain [33].

We estimated the connectivity probability by applying probabilistic tractography at the voxel level using a sampling of 5000 streamline fibres per voxel. Voxels were defined based on the binary brain mask for the whole brain. The connectivity probability from a seed voxel *i* to another voxel *j* was defined by the proportion of fibres passing through voxel *i* that reach voxel *j* [33]. This was then extended from the voxel level to the region level, i.e. in a brain region consisting of *n* voxels, 5000*n fibres were sampled. The connectivity probability *P_ij_* from region *i* to region *j* is calculated as the number of sampled fibres in region i that connect the two regions divided by 5000*n, where *n* is the number of voxels in region *i*.

For each brain region, the connectivity probability to each of the other 91 regions was calculated. It should be noted, that because of the dependence of tractography on the seeding location, the probability from *i* to *j* is not necessarily equivalent to that from *j* to *i*. However, these two probabilities are highly correlated across the brain for all participants (the least Pearson *r* = 0.70, *p*<10^−50^). We therefore defined the undirectional connectivity probability *P_ij_* between regions *i* and *j* by averaging these two probabilities, and considered this as a measure of the structural connectivity between the two areas, with *C_ij_ = C_ji_*. We implemented the calculation of regional connectivity probability using in-house Perl scripts. Regional connectivity was normalised using the regions’ volume expressed in number of voxels. For both pre- and postoperative conditions, a 92×92 symmetric weighted network *C* was constructed, representing the anatomical network organisation of the brain. For all 9 healthy participants a single averaged 92×92 symmetric connectivity network *C* was created.

**Figure 2:**
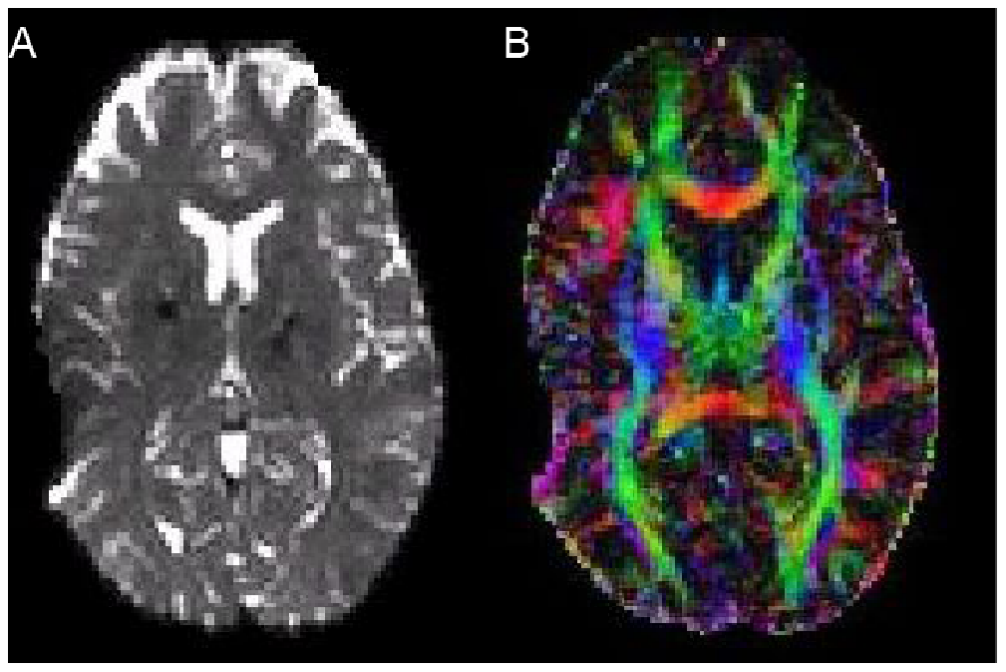
DBS electrode artefact from lead. The artefact from the external lead of the DBS electrodes is visible in the form of dropout in left hemisphere in A) the *b*0 weighted image and B) the diffusion tensor image.

**Figure 3:**
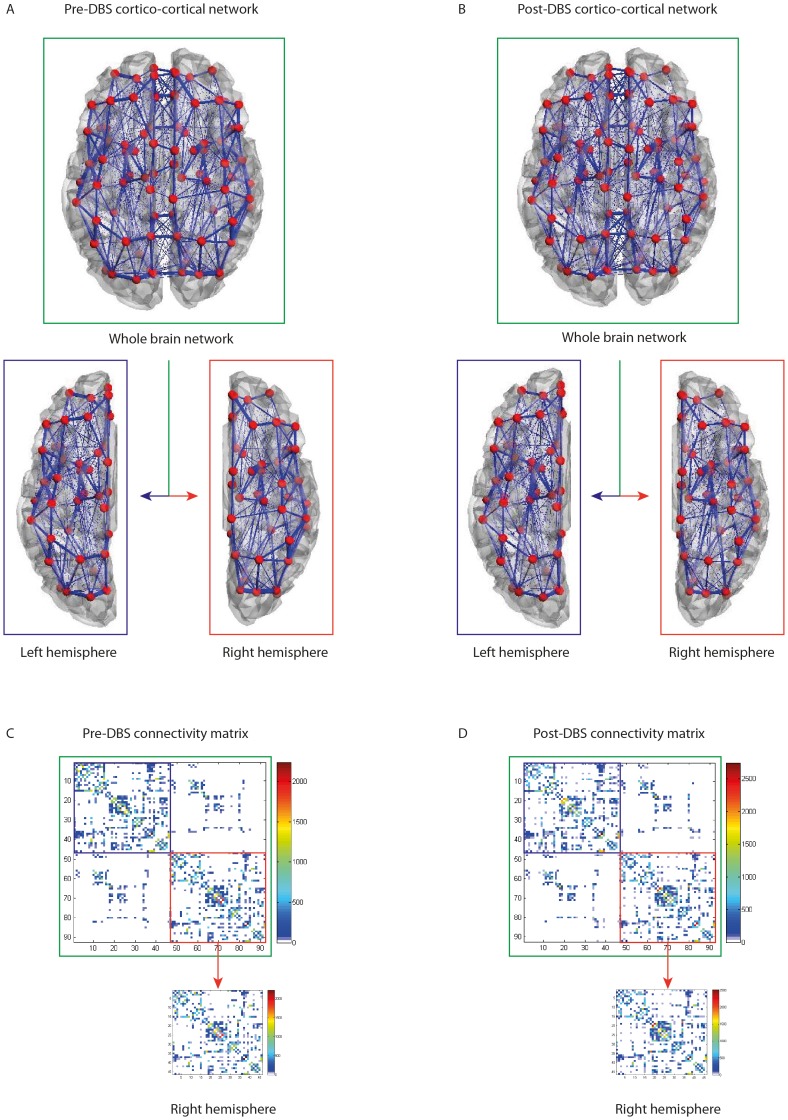
Anatomical connectivity networks derived from DTI data. The pre- and post-operative structural networks (left and right columns, respectively) are shown superimposed on a rendered brain (**A/B**) and as connectivity matrices, *C_pre_* and *C_post_* (**C/D**). In both representations, the full 90-node networks are highlighted in green, while the left and right hemispheres are highlighted in blue and red, correspondingly (In **Table 2** we report the indexing of brain areas). In the matrix representations, transcallosal connectivity is shown in the other diagonals. In C and D, the red arrows point to the pre- and post-operative 45×45 right hemisphere connectivity matrices.

Further to this, as a precautionary measure resulting from the visible postoperative artefact of the DBS lead in the left hemisphere (see **Figure 2**), we considered only the sub-network corresponding to the right hemisphere, i.e. not the full 92×92 connectivity matrix but only the right hemispheric 46×46 matrix (without inter-hemispheric connections) as shown in **Figure 3** [34]. We chose to do so even if the artefact may not be immediately obvious from the connectivity matrix in **Figure 3B**. Subsequent analyses were carried out on the right hemisphere only, also for the healthy connectivity matrix.

### Network Measures from Graph Theory

The structural brain networks derived from DTI can be represented as 46×46 connectivity matrices, *C_preDBS_*, *C_postDBS_* and *C_controls_*, that can be analysed as graphs. Using the Brain Connectivity Toolbox [35], [36], the brain networks were characterized using measures from graph theory. Note that we used the same graph measures used in previous studies of schizophrenia [37], [38].

#### Connection density

The connection density is the number of edges in a network divided by the maximum number of possible edges [(*N^2^* − *N*)/2, where *N* is the number of nodes in the network].

#### Global efficiency

The global efficiency, *E_g_* of a network *C* reflects how efficiently information can be exchanged over that network. The global efficiency is calculated as the average inverse shortest path length, *d_ij_*, and is inversely related to the characteristic path length.
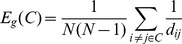
(1)


#### Nodal efficiency

Nodal efficiency, *E_n_*, is used to calculate how efficiently a node is connected to all other nodes in the network. Nodes with high efficiency can reach all other nodes in the network taking, on average, fewer edges. The nodal efficiency is calculated as follows:
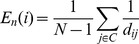
(2)


#### Clustering coefficient

The clustering coefficient (*Cl*) is the fraction of triangles around a node and is equivalent to the fraction of a node’s neighbours that are neighbours of each other.
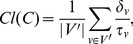
(3)where *δ_v_* is connected triangles, *τ_v_* is connected triples and *V*′ is the set of nodes with degree >2.

#### Small-worldness

A network *C* is considered to be small-world (*σ*>1), if the average shortest path length is small (which is equivalent to having high efficiency *E*) and the clustering coefficient *Cl* is high, when compared to an equivalent random graph *R*. *σ* is calculated as
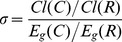
(4)


#### Robustness

When a node is removed, either randomly (random attack) or in order of higher degree (targeted attack), the network can fragment into two or more independent subgraphs. The robustness of a network is estimated by calculating the size of the largest connected component, s, each time a node is removed from the network. The robustness is defined as the area under the *s(n)* curve, where *n* the number of nodes removed [39], normalised by N(N-1)/2. More robust networks retain a larger connected component even when a large proportion of nodes have been eliminated.

#### Hierarchy

The hierarchy coefficient β is the (positive) exponent of the power-law relationship between the clustering C_i_ and the degree d_i_ of the nodes in the network such that C∼d ^−β^ [40], [41]. β was estimated using the least-squares nonlinear fitting function from Matlab®. The higher the hierarchy coefficient, the more network hubs -defined so for having a large degree- have low clustering, meaning that they are more connected to nodes poorly connected to each other.

All global graph measures were calculated for the preoperative, the 5-month postoperative and the average healthy anatomical brain networks. For local measures (such as nodal efficiency) differences between pre- and postoperative measures were only considered if the difference was 20% or above. This conservative decision was based on a test-retest reliability study by Cheng and colleagues [42], who have shown that test-retest variability is below 10% for weighted structural networks derived from DTI.

In addition, we used the same graph measures to characterize the functional networks obtained with the model. However, since the functional connectivity represents the correlation between BOLD signals, we need to define a correlation threshold above which a functional link is considered. To illustrate the differences between pre-DBS and post-DBS we applied a threshold so that any correlation in the connectivity matrix higher than 0 was included and considered as a functional connection in a binarised graph.

### Dynamic Mean Field Model

To gain insight into the impact of the local structural changes induced by DBS in the whole-brain activity, we use a computational model to simulate the spontaneous dynamics of the whole brain. The dynamics of each brain area (constituted by a large number of densely interconnected spiking neurons) can be represented in a reduced manner by its mean field. The classical mean field model [43] is used to calculate the steady states of the spiking network only. However, since we are interested on the temporal dynamics of this mean field (to investigate temporal correlations between brain areas) we use a dynamic mean field model [16], [20] which approximates the temporal dynamics of the spiking network. Brain areas are then coupled together (i.e. receive excitatory inputs from other brain areas) according to the anatomical structural connectome.

In short, the dynamic mean field approximation reduces the spiking network model, including the whole dynamics of each local network of excitatory and inhibitory populations of spiking neurons interconnected by AMPA, GABA and NMDA receptors and their respective equations [44], to a single one dimensional equation [16], [20]. Thus the global brain dynamics, represented as a network of inter-connected local networks, can be simply and consistently described by the following set of coupled differential equations:
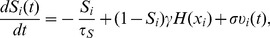
(5)

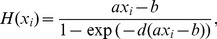
(6)

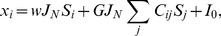
(7)


where 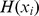 and 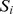 denote the population rate and the average synaptic gating variable at the local cortical area 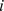 (from 1 to *N* = 45 areas in our case), respectively. *w* = 0.9 is the local excitatory recurrence and 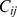 corresponds to the coupling weight between the areas *i* and *j*. Note that 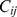 is estimated from the structural connectivity, i.e. in proportion to the number of white-matter tracts detected between areas *i* and *j,* and therefore this parameter is changed between pre-DBS, post-DBS and control data. 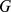 is the global coupling weight that scales all the couplings from 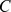 uniformly. Parameter values for the input–output function (6) are 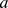 = 270 VnC, 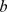 = 108 Hz, and 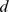 = 0.154 s. The kinetic parameters are 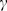 = 0.641/1000 (the factor 1000 is for expressing everything in ms), and 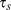 = 100 ms. The synaptic couplings are 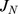 = 0.2609 nA and the overall effective external input is 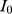 = 0.3 nA. In equation (5) 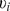 is uncorrelated standard Gaussian noise and the noise amplitude at each node is 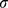 = 0.001 nA.

Simulations were run using the three structural connectivity matrices, i.e. C_preDBS_, C_postDBS_ and C_controls_, and the bifurcation points where the dynamics becomes unstable were identified. The bifurcation analysis uses rigorous simulations and is based on finding the fixed points of the network as a function of the parameters. Long lasting simulations are run until convergences appear and each simulation starts for 1000 different random initial conditions.

Subsequently, the mean field activity was transformed into BOLD signal using the Balloon-Windkessel hemodynamic model of [45] (as in [16], [19]). The Balloon-Windkessel model describes the transduction of neuronal activity into perfusion changes and subsequently into BOLD signal. The BOLD-signal estimation for each brain area is computed by the level of synaptic activity in that particular area, represented by the synaptic variable 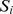. The simulated BOLD signal was down-sampled at 2s to have the same temporal resolution as in the empirically measured BOLD signal. The simulated FC between all brain areas is obtained by computing the temporal correlation matrix of the simulated fMRI signals.

The different FCs simulated with the model (i.e. obtained with C_preDBS_, C_postDBS_ and C_controls_) were compared with an empirical resting FC, provided to us by He and colleagues [21], [46]. This empirical FC matrix represents the average FC obtained from 18 right-handed healthy young volunteers (9 females, age range 21–25 years). The participants were scanned using a 3T GE MR scanner (EXCITE, Milwaukee, USA). The images were obtained using an echo-planar imaging (EPI) sequence with the following parameters: 30 axial slices, slice thickness = 4.5 mm with no gap, matrix = 64×64, TR = 2,000 ms, TE = 30 ms, flip angle = 90°, field of view = 220×220 mm^2^. Participants were instructed to lay completely still, keep their eyes closed and relax their minds as much as possible. See [46] for a full description of the preprocessing of the resting-state fMRI data.

For the functional modelling, only 45 regions from the DTI matrices for the right hemisphere were used. The STN was excluded from this part o the analysis, as data for the STN connectivity was not present in the data from He and colleagues [21], [46]. However, as all matrices from the structural and functional datasets resulted from MNI AAL template parcellation, no further preprocessing steps were necessary as input for the dynamic mean field modelling.

## Results

We started by analyzing the pre- and post-DBS as well as the healthy structural networks built from DTI using measures from graph theory. Please note that, due to the visible DBS lead artefact in the left hemisphere on the post-operative DTI, all measures reported in the following refer to the right hemisphere (see **Figure 3** and *Methods-Structural Brain Networks* for details).

Overall, in terms of global graph measures, there was no apparent shift between pre and post-DBS towards healthy structural connectomes (see **Table 1**). Both pre- and post-operative structural networks exhibit a small-world topology in the right hemisphere as characterized by σ>1. The connection densities for the preoperative and postoperative network were found to be 45.4% and 45.9%, respectively. Contributing to the small-worldness of the network are both high global efficiency and clustering coefficients seen in both preoperative and postoperative networks.

**Table 1:** Graph properties of the preoperative, postoperative and control structural networks (right hemisphere only).

	PD Patient Before DBS	PD Patient After DBS	Healthy controls
**Connection density**	45.0851	45.9357	60.1134
**Average clustering coefficient**	0.6680	0.6955	0.7668
**Global efficiency**	0.7295	0.7341	0.8072
**Small-worldness**	1.1912	1.2118	1.0829
**Hierarchy**	0.1369	0.1233	0.0818
**Robustness (random)**	0.9971	0.9923	0.9990
**Robustness (targeted)**	0.9411	0.8947	0.9372

**Figure 4:**
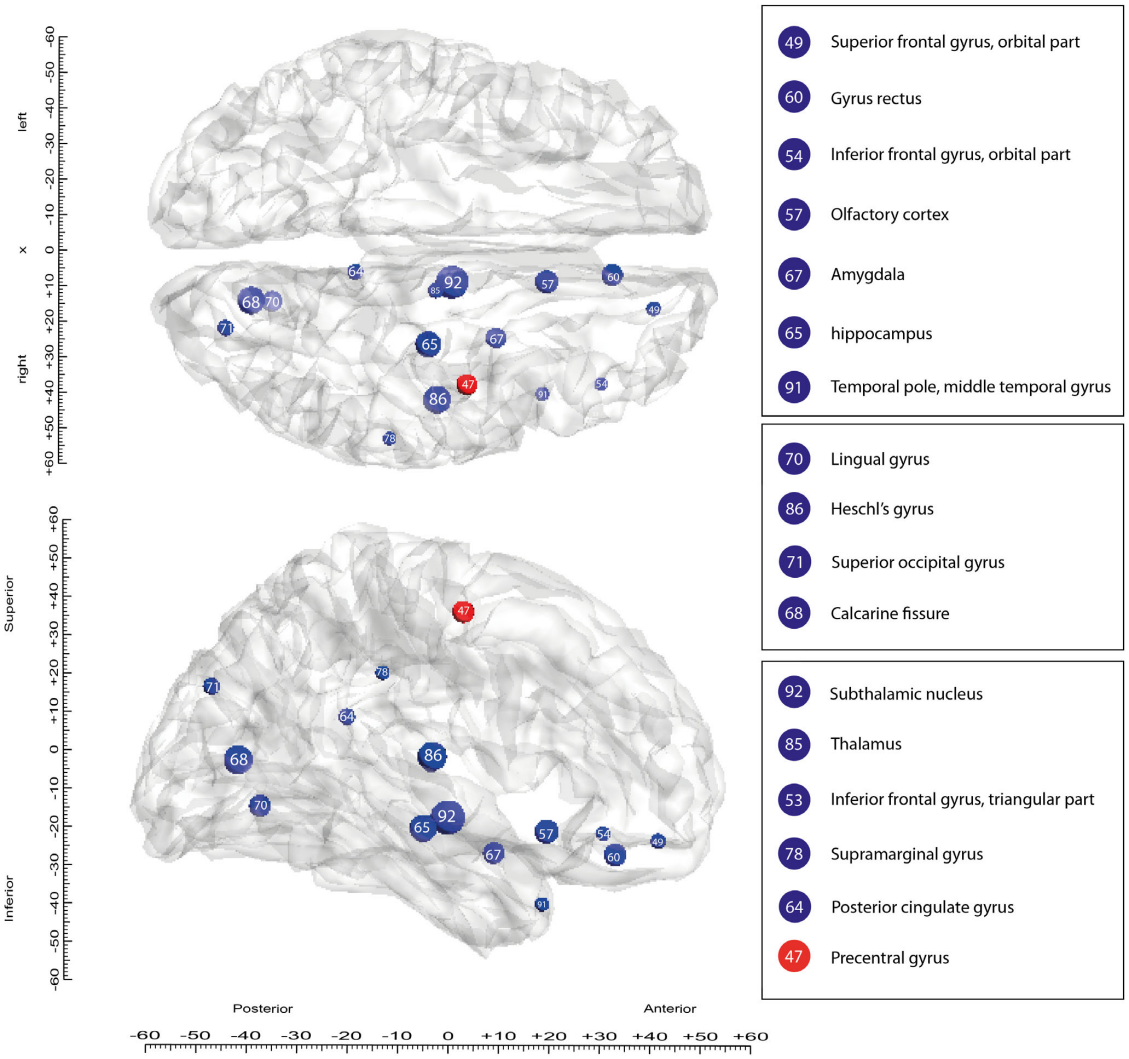
Nodal efficiency changes between pre- and post-DBS structural networks. The AAL regions with more than 20% difference in nodal efficiency between pre- and postoperative measures are plotted on three-dimensional renderings of the human brain in MNI space seen from above (top) and from the side (bottom). The size and colour of the circles indicate the magnitude (size) of the increases (blue) and decrease (red) in nodal efficiency after DBS. The number inside each circle indicates the AAL ordering index reported in **Table 2**.

In contrast, some local graph measures, i.e. referring to specific brain areas, were significantly different between pre- and post-DBS. A total of 17 brain regions (defined in the AAL+STN parcellation scheme) show more than a highly conservative 20% significant difference between preoperative and postoperative nodal efficiency measures. Sixteen of these changes correspond to increases in efficiency after 5 months of DBS, while there was also one decrease. All 46 pre- and post-operative nodal efficiencies for the right hemisphere are shown in **Table 2**, while **Figure 4** shows the size and location of the significant changes induced by DBS in the efficiency of brain regions on a three-dimensional rendering of the brain.

**Table 2:** Listing the 45**Figure 3**.

AAL region	Left	Right	Preoperative nodal efficiency	Postoperative nodal efficiency	Difference (%)
**Precentral gyrus**	1	47	154.83	111.39	**−28.05**
Superior frontal gyrus, dorsolateral	2	48	142.49	163.69	14.87
**Superior frontal gyrus, orbital part**	3	49	97.46	128.07	**31.41**
Middle frontal gyrus	4	50	111.20	124.39	11.86
Middle frontal gyrus, orbital part	5	51	65.53	75.46	15.14
Inferior frontal gyrus, opercular part	6	52	145.84	126.38	**−**13.34
Inferior frontal gyrus, triangular part	7	53	91.04	94.65	3.97
**Inferior frontal gyrus, orbital part**	8	54	95.03	123.60	**30.06**
Rolandic operculum	9	55	224.32	227.93	1.60
Supplementary motor area	10	56	123.85	102.34	**−**17.36
**Olfactory cortex**	11	57	113.32	161.20	**42.26**
Superior frontal gyrus, medial	12	58	111.14	110.65	**−**0.43
Superior frontal gyrus, medial orbital	13	59	126.44	138.98	9.92
**Gyrus rectus**	14	60	109.46	154.57	**41.21**
Insula	15	61	108.30	126.86	17.13
Anterior cingulate and paracingulate gyri	16	62	165.09	194.93	18.08
Middle cingulate and paracingulate gyri	17	63	169.76	174.84	3.00
**Posterior cingulate gyrus**	18	64	129.20	162.06	**25.43**
**Hippocampus**	19	65	112.79	166.70	**47.80**
Parahippocampal gyrus	20	66	110.91	119.84	8.05
**Amygdala**	21	67	113.45	156.20	**37.68**
**Calcarine fissure**	22	68	120.80	178.42	**47.70**
Cuneus	23	69	200.12	207.92	3.90
**Lingual gyrus**	24	70	91.98	135.11	**46.90**
**Superior occipital gyrus**	25	71	139.42	173.18	**24.22**
Middle occipital gyrus	26	72	136.09	130.53	**−**4.08
Inferior occipital gyrus	27	73	109.82	124.72	13.57
Fusiform gyrus	28	74	149.29	157.77	5.68
Postcentral gyrus	29	75	128.16	109.54	**−**14.53
Superior parietal gyrus	30	76	118.85	114.12	**−**3.98
Inferior parietal gyri	31	77	193.01	160.54	**−**16.82
**Supramarginal gyrus**	32	78	135.67	163.88	**20.79**
Angular gyrus	33	79	131.60	136.13	3.44
Precuneus	34	80	136.77	162.83	19.05
Paracentral lobule	35	81	124.05	126.07	1.63
Caudate nucleus	36	82	99.06	105.85	6.85
Putamen (lenticular nucleus)	37	83	102.70	110.43	7.52
Pallidum (lenticular nucleus)	38	84	113.83	127.50	12.00
**Thalamus**	39	85	81.77	114.11	**39.54**
**Heschl’s gyrus**	40	86	222.10	279.70	**25.93**
Superior temporal gyrus	41	87	154.29	174.56	13.13
Temporal pole: superior temporal gyrus	42	88	133.97	138.50	3.38
Middle temporal gyrus	43	89	93.18	92.25	**−**1.00
**Temporal pole: middle temporal gyrus**	44	90	108.33	136.92	**26.38**
Inferior temporal gyrus	45	91	81.71	97.19	18.95
**Subthalamic nucleus**	46	92	96.72	164.80	**70.38**

Pre- and post-operative nodal efficiency measures and corresponding percentage differences are reported. AAL regions identified to have more than 20% difference between preoperative and postoperative nodal efficiency are shown in bold.

**Figure 5:**
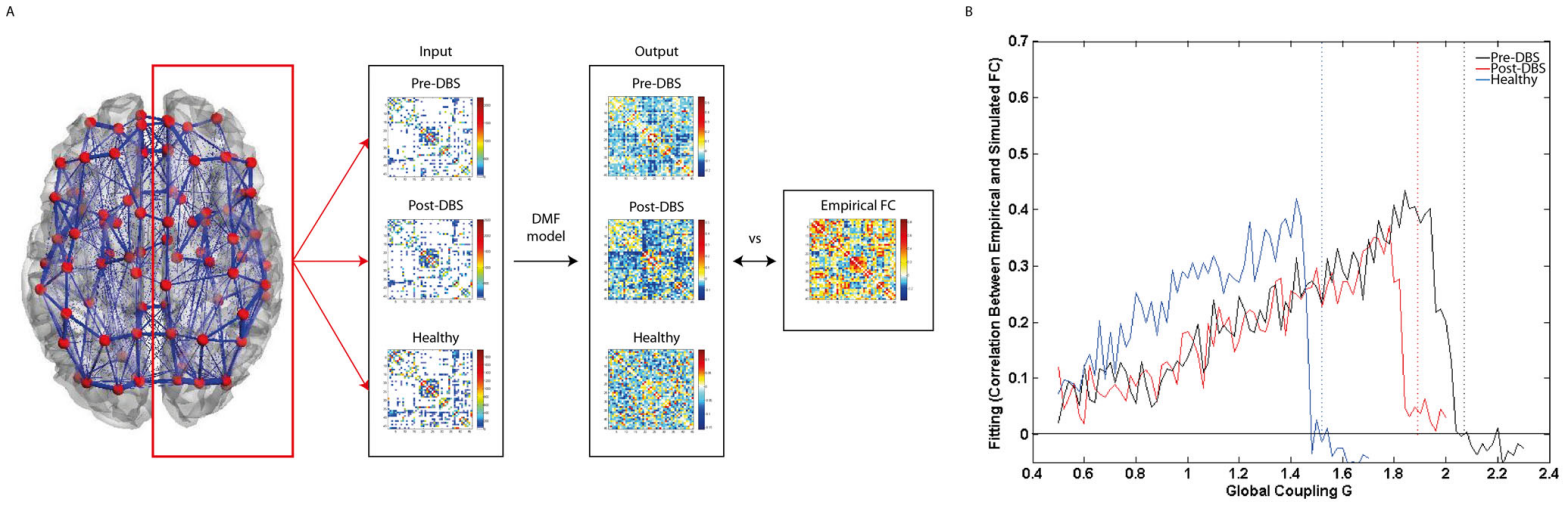
Exploring the impact of DBS-induced structural changes on resting-state functional connectivity. **A**) Schematic overview of how the simulated FC matrices are obtained from the SC matrices using the dynamic mean field (DMF) model. The simulated FC matrices are subsequently compared to the empirical resting state FC matrix. **B**) Solid lines indicate the fitting of simulated functional connectivity (FC) matrices obtained with the pre-DBS (black), post-DBS (red), and healthy controls (blue) structural connectivity matrices with the empirical healthy FC, as a function of the global coupling weight (G). Vertical dashed lines indicate the corresponding bifurcation points, above which the dynamics becomes unstable. We observe that the bifurcation point of the post-DBS FC is shifted from the pre-DBS FC bifurcation point towards the healthy bifurcation point. This means that, before DBS, the structural connectivity is weaker and therefore stronger couplings are required to reach an optimal fitting with empirical FC The shift of the post-DBS bifurcation point towards the healthy bifurcation point indicates recovery of the structural connectivity with DBS.

To study the impact of DBS-induced structural changes in the whole-brain activity, the spontaneous dynamics resulting from the different structural connectomes (i.e. from pre-DBS, post-DBS and healthy controls) was estimated by means of a computational model. As shown in **Figure 5** **,** it was found that the bifurcation point (above which the dynamics becomes unstable) was shifted in the disease. In other words, before DBS in PD, the structural connectivity is weaker and therefore stronger couplings are required to reach the instability border, near which an optimal fitting with empirical FC is obtained. Note that, despite the shift of the bifurcation, the FC exhibits homeostasis, which means that the optimal fit with the empirical data is always found just below the bifurcation threshold, regardless of which connectome is considered. Still, our results show that after 5 months of DBS, the structural connectome is changed in such a way that the bifurcation point is now obtained at a weaker global coupling. This shift of the bifurcation point towards the value of healthy controls indicates recovery of the structural connectivity with DBS.

To gain more insight into the changes occurring at the FC level, the topological properties of the optimal FCs (i.e. corresponding to the maximal fitting of the empirical FC) obtained with the model from pre-DBS, post-DBS and controls structural connectomes were analysed and compared. As shown in **Table 3** **,** the connection density, the average clustering, the global efficiency and the small-worldness of functional networks were increased and the hierarchy was decreased after DBS, in the direction of the values obtained for the control FC.

**Table 3:** Graph properties of simulated functional networks derived from the dynamic mean field model with the 3 structural connectomes from the preoperative PD, postoperative PD and healthy controls.

	Pre-DBS PD	Post-DBS PD	Healthy Controls	Empirical FC
**Connection density**	28.5432	27.9506	30.7160	60.3457
**Average Clustering**	0.4061	0.4185	0.3589	0.6957
**Global efficiency**	0.2919	0.2859	0.3141	0.6172
**Small-worldness**	1.4668	1.4047	1.1678	1.1236
**Hierarchy**	0.3561	0.3499	0.3959	0.1104
**Robustness (Random)**	0.9949	0.9960	0.9808	0.9990
**Robustness (Targeted)**	0.8990	0.8848	0.8889	0.9778

These results indicate that prolonged DBS overall has restorative effects in the structural connectome and furthermore affects the functional connectivity at a global level.

## Discussion

This is the first study investigating the long-term effects of DBS on neuroplasticity in the human brain. We used the rare opportunity to acquire pre- and post-operative *in vivo* neuroimaging data in a patient with PD with DBS in the STN. We employed advanced computational modelling and graph theoretical analyses to study the long-term effects on global and local measures of brain structural and functional connectivity networks.

The patient showed improvement in rigidity from right DBS lead stimulation. The left DBS lead stimulation resulted in a reduction of the tremor on the right side, but was also accompanied by problematic side effects such as facial tightness and after prolonged stimulation emotional lability and tearfulness. Although stimulation improved left sided rigidity and right sided tremor, the DBS stimulation was *on* intermittently due to the adverse side effects. These side effects led to the postoperative DTI data acquisition to plan a DBS lead revision to the GPi.

Overall, in terms of structural connectivity, the results show that DBS gives rise to *local* changes in specific brain regions, as measured by changes in terms of nodal efficiency. However, *global* measures such as small-worldness were unaffected between pre- and post-DBS connectivity. These local changes were found primarily in regions that can be roughly characterised as sensory-motor, prefrontal/limbic and sensory regions.

In terms of functional connectivity, this study also shows that as predicted there is a shift in bifurcation point between pre-DBS and post-DBS networks towards the bifurcation point of a healthy network. This means that the structural connectivity of the pre-DBS network is weaker and requires stronger couplings for the bifurcation. The couplings required for the post-DBS structural connectivity network are lower and thus the bifurcation is closer to the healthy network, which requires the lowest couplings. The shift towards the healthy bifurcation point indicates recovery of some functional connectivity after five months of DBS.

Equally, using dynamic mean field modelling this study shows interesting differences between pre-DBS and post-DBS simulated functional networks. Whereas the structural networks did not show significant differences in global graph theoretical measures between pre- and post-DBS measures, the simulated functional connectivity networks (based on the structural network inputs) show a tendency of global graph theoretical measures to shift towards healthy functional connectivity of post-DBS compared to pre-DBS. Connection density, average clustering (clustering coefficient) and global efficiency show an increase from pre- to post-DBS shifting towards values of the healthy simulation functional network. These findings indicate that the underlying local structural changes induced by DBS influence the functional connectivity on a larger, more global, level.

The structural connectivity changes in regions linked to sensory-motor integration showed mainly decreases in their nodal efficiency post-op, which fits with previous studies showing that STN stimulation alleviates symptoms by modulating the larger thalamocortical loop networks. These networks are involved in setting up, relaying and executing sequential motor program commands [2], [47]. A previous study investigating the functional connectivity of the STN in PD with magnetoencephalography has shown that there is strong coherence between the STN and ipsilateral frontal cortices [5]. One main finding in this study is the decrease in nodal efficiency in the precentral sensory-motor cortical area, while there were increases in the subthalamic nucleus, the thalamus and the supramarginal gyrus. Although these areas are clearly implicated in thalamocortical loops by previous studies, it is interesting to find a structural network change in these areas indicating neural plasticity after prolonged DBS of the STN.

Similarly, increases in nodal efficiency were found in prefrontal/limbic regions which included regions of the olfactory cortex, amygdala, hippocampus, temporal pole and orbitofrontal cortex (OFC). Interestingly, the involvement of most of these areas in olfactory processing has been well established with a reported asymmetry favouring the right OFC [26], [48]. Moreover, olfactory dysfunction is a well-known non-motor symptom in PD and manifests itself in a decrease in behavioural measures such as odour threshold, odour discrimination and odour identification [22], [49]. In addition to these well-known olfactory areas, functional imaging studies on olfactory processing in PD reveal significant differences in the amygdala and hippocampus [50], [51], two areas which also show a large increase in nodal efficiency in this case study after DBS. Furthermore, a DTI study on olfactory dysfunction in PD has shown that the gyrus rectus part of medial OFC shows reduced fractional anisotropy (FA) values of white matter tracts in patients with anosmia or severe microsmia, but not in patients without olfactory dysfunction [52]. These large increases in nodal efficiency in known olfactory areas might point towards changed olfactory functioning after long term DBS. This corresponds to the significant increase in odour discrimination in PD patients with DBS *on* compared to *off* stimulation [25].

Increases in nodal efficiency were also found in sensory regions including visual and auditory cortices, with changes in calcarine fissure and surrounding cortex and the superior occipital gyrus, as well as the lingual gyrus and Heschl’s gyrus. This could be linked to a normalisation of sensory systems with DBS given that visual and auditory hallucinations and other sensory impairments are common in PD [53]. At this point, however, it is unknown whether these structural changes reflect functional changes in sensory systems and calls for more research.

Although alleviation of motor symptoms is the main objective, it is well established that DBS of the STN has wide spread effects and influences the majority of non-motor symptoms [54]. Even though no information on the olfactory function of this patient is available, the findings in this study do corroborate previous findings [25]. Furthermore, prefrontal and orbitofrontal areas known to be involved in mood (especially depression) also show considerable changes in this patient and could be argued to explain the adverse side effects in mood lability.

In terms of supporting evidence from animal studies of neuroplasticity following DBS, one recent animal study showed increased new cell survival in the rostral migratory stream, including the hippocampus, and the olfactory bulb [12]. These finding are of great importance for a number of factors including the preclinical manifestation of olfactory dysfunction as well as its high prevalence rate. Additionally, olfactory dysfunction is highly correlated with depression and low quality of life [55] and depression often occurs in PD alongside olfactory dysfunction [56].

A potential limitation of this study is the fact that it only focuses on the right hemisphere, because of the artefact that occurs due to the external DBS lead. Although the results clearly show neural plasticity and network changes after long term DBS in the right hemisphere, inter-hemispheric connections could contain valuable information, especially in bilateral DBS of the STN.

Another limitation is that this study is a single case study, yet the rarity of neuroimaging pre- and post-DBS offers potentially unique insights. The results demonstrate that connectivity measures and network analysis are useful methods to investigate the effects of long term DBS in patients *in vivo*. This can thus be applied on a larger scale and over longer periods of time. More advanced DBS electrodes with less or no lead artefact will increase the possibilities of graph theory analysis on complex brain networks including both hemispheres and transcallosal connections.

Though the DBS lead has been subtracted from the pre-DBS data to minimise any effect of the electrode on the connectivity, the presence of the electrode may artificially alter the connectivity due to the probabilistic nature of the tractography algorithm. Ideally the findings in the current manuscript would have to be compared to post-mortem measures.

The functional modelling is partly based on the data from the patient and partly uses a young healthy control group. It should be noted that the healthy control data is not used in the comparison of pre-DBS and post-DBS connectivity measures. The results clearly indicate that the bifurcation of the functional network shows a shift back towards a healthy regime, however this healthy regime is based on a young control group. Additionally, a large study of 80 subjects investigating the effects of ageing on diffusion weighted imaging has found no significant differences between age groups in the absolute apparent diffusion coefficient [57]. Another study investigating the effects of age on diffusivity and fractional anisotropy actually has a cut off age for the young group at age 47 [58], whereas our patient is only 45 years old.

Furthermore it should be noted that although structural changes are found after DBS, resulting in a change in the functional connectivity, these functional changes may only be apparent when stimulation of the STN is ongoing. I.e. These structural changes may not be fully utilised when stimulation is turned off, thus not showing any long term effects of symptom changes off-stimulation.

In conclusion, we have demonstrated that long term DBS for PD leads to significant changes in local structural connectivity as well as global functional connectivity. The changes were seen in regions previously associated with pathological changes in PD and suggest that DBS helps to re-balance the networks, not only over the short term but in terms of neuroplasticity. In particular, the observed changes in olfactory systems suggest that DBS may help patients to regain some olfactory function. The importance of the extent of this change in olfactory function remains to be investigated in detail.
